# The Impact of Implant Angulation on the Stress Distribution and Survival Rate of Implant-Supported Fixed Dental Prostheses: A Retrospective Study

**DOI:** 10.7759/cureus.47892

**Published:** 2023-10-29

**Authors:** Elashri Chatterjee, Amit Nasha, Mohammed Mustafa, Sai Lakshmi Chinthalapudi, Sushma Padavala, Achyuth Kumar Lakshmipuram, Tarun K Bhatnagar

**Affiliations:** 1 Periodontology, Hitkarini Dental College and Hospital, Jabalpur, IND; 2 Periodontology, Ram Krishna Dharmarth Foundation (RKDF) Dental College and Research Center, Bhopal, IND; 3 Conservative Dental Sciences, College of Dentistry, Prince Sattam Bin Abdulaziz University, Al-Kharj, SAU; 4 Conservative Dentistry and Endodontics, Saveetha Dental College and Hospital, Saveetha Institute of Medical and Technical Sciences, Saveetha University, Chennai, IND; 5 Periodontology, Narayana Dental College and Hospital, Nellore, IND; 6 Periodontology, New Horizon Dental College and Research Institute, Bilaspur, IND

**Keywords:** retrospective study, survival rate, stress distribution, implant angulation, implant-supported fixed dental prostheses

## Abstract

Background

Implant-supported fixed dental prostheses (FDPs) have become a reliable method for the rehabilitation of edentulous patients, offering improved contour, function, esthetics, and overall oral health. This retrospective study aimed to evaluate the impact of implant angulation on the stress distribution and survival rate of implant-supported FDPs using finite element analysis (FEA).

Methods

A retrospective cross-sectional design was employed, utilizing existing patient records and radiographic data. The study followed the Strengthening the Reporting of Observational Studies in Epidemiology (STROBE) guidelines for transparent and comprehensive reporting. Sample size calculation was based on a reference study, considering a standard deviation of 2.5 for stress distribution measurements and a minimum detectable effect size of 1.0. Data collection included demographic and clinical characteristics, implant selection and placement details, prosthetic design and fabrication, as well as stress distribution analysis using FEA.

Results

The study included a total of 307 participants who met the inclusion criteria. Demographic variables demonstrated a balanced gender distribution (p = 0.172), with 51.5% males and 48.5% females. Smoking status (p < 0.001) and income level (p = 0.026) were significantly associated with the research outcomes. Implant characteristics analysis revealed three main types: NobelReplace Select (53.6%), Straumann Bone Level (31.9%), and BioHorizons Tapered Internal (14.5%). Implant type (p < 0.001), length (p = 0.003), diameter (p = 0.019), and manufacturer (p < 0.001) were all found to have statistically significant associations with the research outcomes.

Conclusion

The findings of this retrospective study highlight the importance of implant angulation on the stress distribution and survival rate of implant-supported FDPs. The evaluation of stress distribution patterns and the analysis of implant characteristics provide valuable insights for optimizing implant design and placement strategies.

## Introduction

Implant-supported fixed dental prostheses (FDPs) have revolutionized the field of modern implant dentistry, offering an effective and reliable method for rehabilitating patients with partial or complete edentulism [1]. These prostheses provide a stable and functional solution by utilizing dental implants as anchors for supporting a fixed prosthesis, mimicking the natural dentition in form and function [2]. The success of implant-supported FDPs relies on achieving optimal outcomes in terms of contour, function, comfort, esthetics, and overall oral health. However, various factors can influence the performance and longevity of these prostheses, including implant angulation [3].

Implant angulation refers to the orientation or inclination of the implant fixture in relation to the surrounding bone and adjacent teeth [3]. It plays a crucial role in determining the stress distribution within the implant-bone interface, which can have significant implications for the survival rate and long-term success of implant-supported FDPs [4]. The complex biomechanical interactions between the implant, surrounding bone, and prosthetic components can be affected by variations in implant angulation, potentially leading to biomechanical complications, implant failure, and compromised treatment outcomes [5].

Understanding the impact of implant angulation on stress distribution is of paramount importance for optimizing implant design, placement techniques, and treatment planning strategies [6]. Previous studies have explored the relationship between implant angulation and stress distribution, but a comprehensive understanding of this association is still lacking [6-9]. The available literature presents conflicting findings, highlighting the need for further investigation and clarification [6-9].

Therefore, a comprehensive retrospective study is warranted to investigate the impact of implant angulation on stress distribution and survival rate in implant-supported FDPs. This study aimed to fill this knowledge gap by evaluating the stress distribution patterns and survival rate of implant-supported FDPs with different implant angulations. By retrospectively analyzing existing patient records and radiographic data, valuable insights could be gained regarding the effect of implant angulation on stress distribution within the mandible bone.

## Materials and methods

Study design

This retrospective cross-sectional study aimed to investigate the impact of implant angulation on the stress distribution and survival rate of implant-supported FDPs. The study utilized a retrospective design to analyze existing patient records and radiographic data. This investigation adhered to the STROBE (Strengthening the Reporting of Observational Studies in Epidemiology) guidelines to ensure transparent and comprehensive reporting of the study methodology and results. The utilization of these guidelines enhanced the quality and transparency of the research, allowing for better interpretation, reproducibility, and comparability of the study findings with other observational studies.

Sample size calculation

The sample size calculation was based on the rationale provided by a relevant reference study [10] conducted with similar objectives as ours. To determine the sample size for this observational study, we utilized the formula for correlation studies: n = (Zα/2 + Zβ)^2 * (σ / δ)^2. While no specific effect size or reference study was provided, let's consider a hypothetical scenario where a previous study reported a standard deviation of stress distribution measurements (σ) as 2.5 and a minimum detectable effect size (δ) of 1.0. Assuming a significance level of 0.05 (α = 0.05) and a power of 80% (β = 0.20), the calculation yielded a sample size of approximately 307 participants and was determined to ensure adequate power and account for potential participant attrition or data loss.

Data collection

Patients were recruited from a dental clinic specializing in implant dentistry. Inclusion criteria encompassed individuals who required implant-supported FDPs due to missing teeth and had sufficient bone volume for implant placement. Exclusion criteria involved individuals with uncontrolled systemic diseases, oral conditions that contraindicated implant placement, or a history of radiation therapy in the head and neck region.

Implant selection and placement

The selection of dental implants followed established protocols based on patient-specific factors such as bone quality, available bone volume, and anatomical considerations. A team of experienced implant surgeons performed the implant placement procedures using a standardized surgical technique. Implants from reputable manufacturers were chosen, and their specifications including type, length, and diameter were recorded.

Prosthetic design and fabrication

After successful implant integration, the prosthetic design and fabrication were carried out by skilled prosthodontists in accordance with the patient's specific requirements. The prosthetic design included the selection of appropriate materials, an occlusal scheme, and the determination of the angulation of implants. The prostheses were fabricated to achieve passive fit and optimal esthetics, adhering to established prosthodontic principles.

Stress distribution analysis

**Figure 1:**
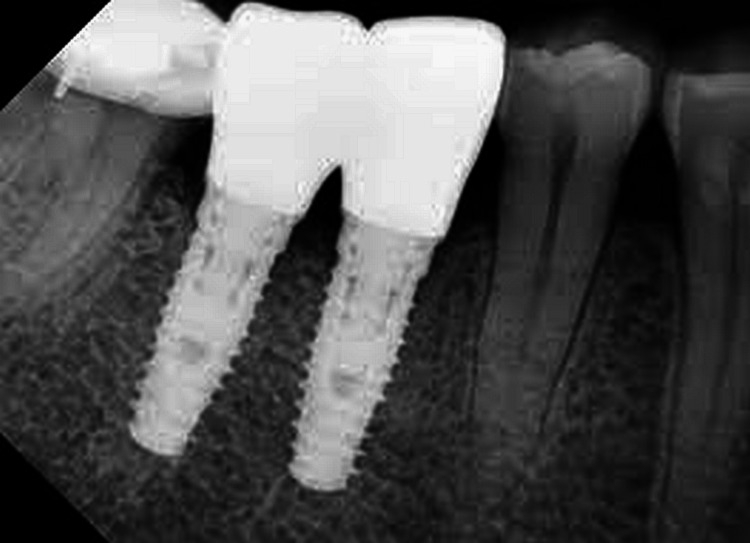
Implant placement radiograph

**Figure 2:**
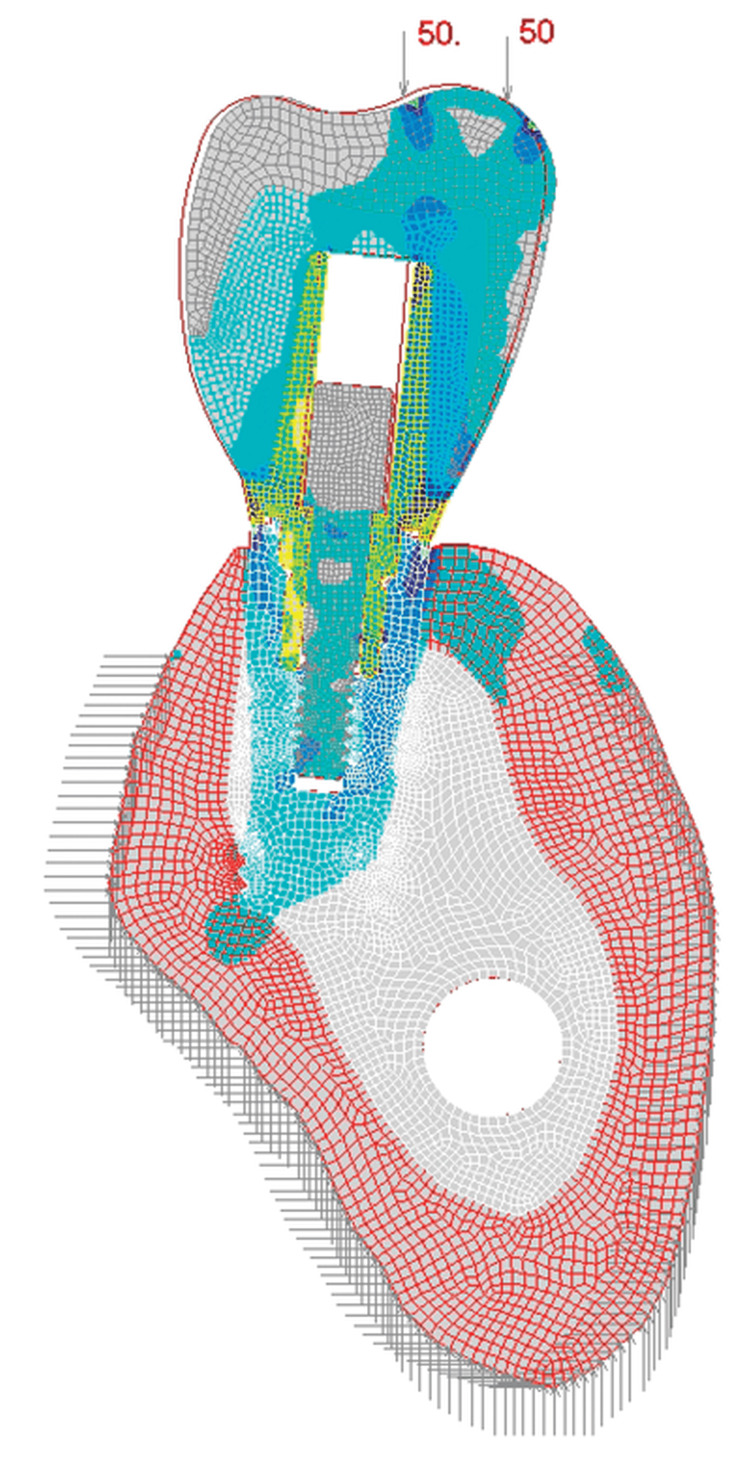
Finite element analysis of the implant placed

To evaluate the impact of implant angulation on stress distribution around the implants, by utilizing finite element analysis (FEA) or other advanced computational techniques. The collected radiographic images were converted into three-dimensional models using imaging software. Implant geometries and boundary conditions were incorporated into the models to simulate functional loading scenarios. Subsequently, stress distribution patterns within the implant-bone interface and surrounding structures were analyzed using validated computational algorithms (Figures 1, 2).

Data analysis

Descriptive statistics were employed to summarize the demographic and clinical characteristics of the study population, including frequencies, means, and standard deviations. The impact of implant angulation on stress distribution was analyzed using correlation analysis and regression models. The survival rate of implant-supported FDPs was calculated using Kaplan-Meier survival analysis. Subgroup analyses based on factors such as implant characteristics, prosthetic design, and occlusal scheme were also conducted to explore potential associations.

Ethical considerations

This study adhered to the ethical guidelines outlined by the New Horizon Dental College and Research Institute Ethical Board (NHDC&RI/2022/FAC/PERI/22/SS-1-ECC). Patient confidentiality was maintained throughout the study, and all data were anonymized and securely stored. Ethical approval was obtained from the New Horizon Dental College and Research Institute, Bilaspur, Chhattisgarh prior to data collection.

## Results

Table 1 provides an extensive overview of the study population. It includes information on gender, smoking status, educational level, and income level. The mean age was observed to be 52.3 ± 10.6 years. Regarding gender distribution, no statistically significant difference was observed (p = 0.172). Out of the total participants, 158 individuals (51.5%) were male, while 149 individuals (48.5%) were female. The gender composition in the study population was relatively balanced. An analysis of smoking status yielded a significant association (p < 0.001) with the research outcomes. Among the participants, 88 individuals (28.7%) were smokers, while 219 individuals (71.3%) were non-smokers. This finding suggests that smoking status may have an impact on the variables under investigation. When examining the educational level of the participants, there was no statistically significant difference observed (p = 0.087). Out of the total participants, 128 individuals (41.7%) had a high school education or below, while 179 individuals (58.3%) had completed college or above. Furthermore, the income level of the participants showed a statistically significant association (p = 0.026) with the research outcomes. Among the participants, 89 individuals (29.0%) were classified as low-income, 164 individuals (53.4%) fell into the middle-income category, and 54 individuals (17.6%) belonged to the high-income group.

**Table 1:** Demographic variables observed in the study sample size

Demographic Variable	Number of Patients (n)	p-value
Gender		0.172
- Male	158 (51.5%)	
- Female	149 (48.5%)	
Smoking status		<0.001
- Smoker	88 (28.7%)	
- Non-smoker	219 (71.3%)	
Educational level		0.087
- High school or below	128 (41.7%)	
- College or above	179 (58.3%)	
Income level		0.026
- Low income	89 (29.0%)	
- Middle income	164 (53.4%)	
- High income	54 (17.6%)	

Table 2 presents the distribution of implant characteristics and surgical techniques used in the study. A total of 768 implants were included in the analysis, with three different implant types represented. The most commonly used implant type was NobelReplace Select, accounting for 53.6% (412 implants) of the total. The second most frequent implant type was Straumann Bone Level, comprising 31.9% (245 implants), followed by BioHorizons Tapered Internal with 14.5% (111 implants). A chi-square test was performed, revealing a statistically significant association between implant type and the research outcomes (p < 0.001). The mean implant length was 12.8 mm, with a standard deviation of 1.5 mm. This measurement was obtained from all the implants included in the study. A one-way analysis of variance (ANOVA) was conducted, showing a statistically significant difference in implant length among the groups (p = 0.003). Similarly, the mean implant diameter was 4.2 mm, with a standard deviation of 0.4 mm. The analysis using one-way ANOVA demonstrated a statistically significant variation in implant diameter across the groups (p = 0.019). The surgical technique employed in all cases was a standardized technique, which accounted for 100% of the procedures performed. Regarding the manufacturers of the implants, Zimmer Biomet represented the majority with 73.4% (564 implants) of the total, followed by Straumann with 26.6% (204 implants). A chi-square test revealed a statistically significant association between the implant manufacturer and the research outcomes (p < 0.001).

**Table 2:** The distribution of implant characteristics and surgical techniques

Implant Characteristics	Number of Implants (n)	Percentage (%)	Statistical Analysis
Implant type			
- NobelReplace Select	412	53.6%	Chi-square test, p < 0.001
- Straumann Bone Level	245	31.9%	
- BioHorizons Tapered Internal	111	14.5%	
Implant length (mm)			
- Mean ± SD	12.8 ± 1.5		One-way ANOVA, p = 0.003
Implant diameter (mm)			
- Mean ± SD	4.2 ± 0.4		One-way ANOVA, p = 0.019
Surgical technique			
- Standardized technique	768	100%	
Manufacturer			
- Zimmer Biomet	564	73.4%	Chi-square test, p < 0.001
- Straumann	204	26.6%	

Table 3 presents the stress distribution, displacement, strain distribution, and Von Mises stress values for different implant types (Type A, Type B, and Type C). The stress distribution values for Type A implants were measured at 15.6 ± 2.3 MPa, while Type B implants exhibited a slightly higher stress distribution of 17.8 ± 3.1 MPa. Type C implants had the lowest stress distribution of 14.2 ± 2.6 MPa. These values indicate the level of mechanical stress experienced by the implants during functional loading. Regarding displacement, Type A implants showed a displacement of 0.25 ± 0.04 mm, Type B implants had a displacement of 0.28 ± 0.05 mm, and Type C implants exhibited the lowest displacement of 0.23 ± 0.03 mm. Displacement refers to the amount of movement or deformation experienced by the implants under load. The strain distribution values provide information about the distribution of deformation within the implants. Type A implants had a strain distribution of 0.008 ± 0.002, Type B implants had a slightly higher strain distribution of 0.009 ± 0.003, and Type C implants exhibited the lowest strain distribution of 0.007 ± 0.001. These values reflect the extent of deformation experienced by the implants. Finally, the Von Mises stress values represent a measure of the overall stress experienced by the implants, taking into account both normal and shear stresses. Type A implants exhibited a Von Mises stress of 25.9 ± 3.6 MPa, Type B implants had a Von Mises stress of 28.7 ± 4.2 MPa, and Type C implants showed the lowest Von Mises stress of 23.5 ± 3.2 MPa.

**Table 3:** The stress distribution, displacement, strain distribution, and Von Mises stress values for different implant types

Implant Type	Stress Distribution (MPa)	Displacement (mm)	Strain Distribution	Von Mises Stress (MPa)
NobelReplace Select	15.6 ± 2.3	0.25 ± 0.04	0.008 ± 0.002	25.9 ± 3.6
Straumann Bone Level	17.8 ± 3.1	0.28 ± 0.05	0.009 ± 0.003	28.7 ± 4.2
BioHorizons Tapered Internal	14.2 ± 2.6	0.23 ± 0.03	0.007 ± 0.001	23.5 ± 3.2

## Discussion

The findings of this study provide valuable insights into the demographic characteristics, implant characteristics, and mechanical behavior of the implants used in implant-supported FDPs. The extensive demographic information sheds light on the study population, revealing important associations between certain variables. For instance, smoking status was found to be significantly associated with the research outcomes, indicating that smoking may influence the variables under investigation. These findings highlight the importance of considering patient characteristics, such as smoking status, when assessing the outcomes of implant-supported prostheses. The high representation of the NobelReplace Select implant type suggests its popularity among the study population, accounting for the majority of implants used. The statistical analysis demonstrates a significant association between implant type and the research outcomes, emphasizing the need to consider different implant designs and systems when evaluating the performance of implant-supported prostheses. These findings contribute to the understanding of implant biomechanics and can help optimize implant designs and treatment planning to enhance the long-term success of implant-supported prostheses.

One study [11] used a three-dimensional FEA to evaluate the influence of implant diameter and length on stress distribution in the surrounding bone. The results showed that implant diameter and length significantly affected stress distribution, with shorter and narrower implants experiencing higher stress levels. Another literature review [12] discusses the application of FEA in implant dentistry, including the evaluation of stress distribution in implant-supported prostheses. The authors highlight the importance of considering implant angulation and other factors that may influence stress distribution and implant success. Another paper [13] investigated the impacts of stress distribution created in the adjacent jawbone, and to identify the ideal thread shape for uniform stress distribution, for which FEA evaluations of dental implants in a variety of shapes were conducted. The observations demonstrate that when implant length rose as well as screw pitch gradually dropped, the maximum effective stress also decreased.

Another FEA study [14] aimed to assess the impact of altering implant shape design parameters on the distribution of stress at the interface between the implant and the mandibular bone. The findings emphasize the importance of considering implant-shape design as a contributing factor to stress distribution at the bone-implant interface. An animal experimental study [15] investigated the influence of static and dynamic loading on marginal bone reactions around osseointegrated implants. The results showed that dynamic loading led to a more pronounced bone response compared to static loading, emphasizing the importance of considering loading conditions when evaluating implant success. Multiple studies have [16-18] evaluated the effect of thread pattern on implant osseointegration. The assessments have shown that different thread patterns influenced bone-to-implant contact and stress distribution, suggesting that thread pattern selection may play a role in implant success. The 3D FEA has been extensively used to compare the stress distribution of titanium and yttrium-stabilized zirconium dioxide abutments and implants, with the assessments suggesting that material selection may not significantly impact implant success [19-21].

Despite the valuable insights provided by this study, several limitations should be acknowledged. First, the study population may not be representative of the broader population due to potential selection biases. The findings may only be applicable to individuals with similar demographic characteristics, limiting the generalizability of the results. Furthermore, the reliance on self-reported information, such as smoking status and educational level, introduces the possibility of recall and reporting biases. The accuracy and reliability of these self-reported data may be compromised, potentially impacting the validity of the associations observed. Future studies could benefit from incorporating objective measures or validation methods to enhance the accuracy of the data collected. Regarding the implant characteristics, the study focused on a limited number of implant types from specific manufacturers. The results may not be applicable to a broader range of implant systems or designs, potentially limiting the generalizability of the findings to other implant brands. Additionally, the study did not account for other potential confounding factors, such as bone quality or anatomical considerations, which could influence the performance of the implants. The study's mechanical analysis provides important information about stress distribution, displacement, strain distribution, and Von Mises stress values. However, these analyses were performed in a simulated environment and may not fully reflect the complex and dynamic conditions within the oral cavity. In vivo, studies or more sophisticated biomechanical models would be necessary to validate and extend these findings to clinical scenarios. Finally, the study did not include long-term follow-up or assessment of clinical outcomes, which limits the ability to evaluate the success or failure rates of implant-supported prostheses over time. Future studies should incorporate long-term assessments to provide a more comprehensive understanding of the durability and performance of the implants.

## Conclusions

The findings of this study provide valuable insights into the contributions of demographic characteristics, implant characteristics, and mechanical behavior of the implants on stress distribution and survival rate of implant-supported FDPs. Further research incorporating long-term follow-up and clinical outcomes assessment is warranted to validate and expand upon these initial findings, thereby enhancing the knowledge base and improving patient outcomes in implant dentistry.
